# Therapeutic effects of Eucommia ulmoides extract on osteoporosis rat models: a systematic review and meta-analysis

**DOI:** 10.3389/fphar.2025.1619687

**Published:** 2025-09-22

**Authors:** Zhen Chen, Chuan Leng, Tang Rui, Chaoqun Feng, Tong Li, Yang Yu, Lei Zhong, Xiaohong Fan

**Affiliations:** Department of Orthopedics, Hospital of Chengdu University of Traditional Chinese Medicine, Chengdu, China

**Keywords:** Eucommia ulmoides oliv, osteoporosis, bone mineral density, meta-analysis, rats

## Abstract

**Background:**

Eucommia ulmoides Oliv. Is a plant in the family Eucommiaceae and genus Eucommia. For thousands of years, it has been one of the most frequently used botanical medicines. Recent research has highlighted the therapeutic effects of its extracts for osteoporosis. However, its benefits still need to be thoroughly analyzed.

**Objective:**

This study aimed to systematically evaluate the efficacy of *Eucommia ulmoides extract* in osteoporotic rat models and explore its underlying mechanisms.

**Methods:**

Following the PRISMA guidelines, a comprehensive literature search was conducted across PubMed, Web of Science, Embase, and four other databases. A total of 511 records were identified, and 18 randomized controlled trials (RCTs) were ultimately included. The risk of bias in the included studies was assessed using the SYRCLE tool. Data synthesis and statistical analyses were performed using Stata SE 18 and RevMan 5.4 software.

**Results:**

*E. ulmoides extract* significantly improved bone mineral density (SMD = 2.44, 95% CI 1.83–3.05, p < 0.000001), trabecular number (MD = 0.87, 95% CI 0.59–1.15, p < 0.000001), trabecular thickness (MD = 0.02, 95% CI 0.01–0.03, p < 0.000001), and bone volume fraction (SMD = 2.82, 95% CI 1.76–3.88, p < 0.000001), while reducing trabecular separation and structural model index. Serum estradiol levels increased significantly, while tartrate-resistant acid phosphatase and osteocalcin levels decreased. Sensitivity analysis confirmed the robustness of the findings, with no significant publication bias detected.

**Conclusion:**

*E. ulmoides extract* is an effective treatment for osteoporosis. It promotes bone formation, inhibits bone resorption, and improves bone microarchitecture. These findings support its potential as a plant-derived therapeutic agent for osteoporosis.

**Systematic review registration:**

https://www.crd.york.ac.uk/PROSPERO/, identifier CRD420251003546.

## Introduction

Osteoporosis (OP) is a systemic skeletal disorder of multifactorial origin, characterized by decreased bone mineral density, reduced bone quality, and microarchitectural deterioration of bone tissue, leading to significantly increased bone fragility and a heightened risk of pathological fractures (Aibar-Almazán et al., 2022; Ensrud and Crandall, 2024). With the rapid progression of global population aging, the prevalence of osteoporosis has been steadily increasing (Keshishi et al., 2021). Global epidemiological data indicate that between 1990 and 2019, the number of deaths attributable to low bone mineral density (LBMD) increased markedly from 207,367 to 437,884, representing a total growth of 111.16%. During the same period, the Disability-Adjusted Life Years (DALYs) associated with LBMD rose from 8,588,936 to 16,647,466, reflecting an increase of 93.82%. Of particular concern, the disease burden associated with LBMD-related fractures has exhibited a steeper increase: deaths surged from 121,248 to 301,482 cases (an increase of 148.65%), while DALYs soared from 4,436,789 to 9,808,464 person-years (an increase of 121.07%). Furthermore, over the past three decades, the burden of LBMD-related diseases has approximately doubled, with the growth rate of fracture complications exceeding that of the overall LBMD burden by 36.49 percentage points (Shen et al., 2022; Yu and Xia, 2019).

Fractures, the most serious complication of osteoporosis (OP), not only impair physical function and reduce quality of life but also impose a substantial public health and economic burden (Compston et al., 2019). Clinical studies have indicated that fractures occurring at the hip and spine due to site-specific reductions in bone mineral density are classified as typical osteoporotic fractures. Epidemiological data reveal that in 2019, there were 9.58 million new cases of hip fractures globally among individuals aged 55 years and above, representing a 159.75% increase compared with 1990. Among these, 6.2 million new cases occurred in women (an increase of 152.16%) and 3.38 million in men (an increase of 174.95%) (Feng J. N. et al., 2024). Research data show that the median direct medical cost associated with hospitalization for a single hip fracture was 10,075 US dollars (GBD 2019 Fracture Collaborators, 2021).

In the diagnosis of osteoporosis, a comprehensive assessment integrating microscopic parameters of both the trabecular and cortical bone systems is essential. Among these, Bone Mineral Density (BMD), serving as a core indicator of bone mineral content and strength (Seeger, 1997), is measured by Dual-energy X-ray Absorptiometry (DXA) and established as the diagnostic gold standard (Chen et al., 2024). According to the WHO definition, the diagnostic criterion for osteoporosis is a BMD value 2.5 standard deviations (SD) or more below the mean for healthy, gender-matched young adults (Dimai, 2017). Bone biochemical markers dynamically reflect the state of bone remodeling and are crucial for early diagnosis as well as therapeutic efficacy evaluation (Feng X. J. et al., 2024). Among bone formation markers, serum Alkaline Phosphatase (ALP) activity indicates osteoblast function (Chen et al., 2025), Osteocalcin (OC) reflects the level of bone turnover (Ling et al., 2016), and Type I Procollagen Amino-terminal Propeptide (P1NP) characterizes the rate of collagen synthesis. Bone resorption indicators such as serum calcium and phosphorus concentrations participate in mineralization regulation: calcium imbalance suggests metabolic abnormalities, while phosphorus fluctuations may indicate renal phosphate metabolism disorders or potential hyperparathyroidism. Continuous monitoring of these markers provides the basis for precise diagnosis and management of osteoporosis.

Of particular concern is that osteoporosis exhibits chronic progression and a prolonged disease course, necessitating long-term or even lifelong management in clinical practice (Compston et al., 2019). The prevention and treatment of osteoporosis require a multifaceted approach, encompassing basic treatment, pharmacological therapy, lifestyle modifications, and physical therapy. In the preventive management of osteoporotic fractures, pharmacological therapy plays a pivotal role; however, its potential risk of adverse effects has garnered increasing clinical concern (Erviti et al., 2017). Bisphosphonates, currently the most widely prescribed antiresorptive agents for the management of osteoporosis (Khosla and Hofbauer, 2017; Reid and Billington, 2022), have been increasingly associated with severe adverse events such as atypical femoral fractures (AFF) (Shane et al., 2014) and osteonecrosis of the jaw (ONJ) (Khosla et al., 2007) during long-term use.

Traditional Chinese Medicine (TCM), as a major branch of traditional medicine, has a long-standing history of application in the prevention and treatment of osteoporosis (Zhuo et al., 2022). *Eucommia ulmoides Oliver*, a plant belonging to the monotypic genus Eucommia within the Eucommiaceae family, is an endemic relict species native to China, with a medicinal history dating back nearly two thousand years (Wang et al., 2019). In clinical practice, *E. ulmoides Oliver* has been extensively utilized in the treatment of osteoporosis and has shown promising clinical effects (Huanping et al., 2021; Wenyuan et al., 2025). Recent advances in research indicate that several studies have conducted systematic evaluations of the anti-osteoporotic effects of *E. ulmoides extracts*.

Total flavonoids from *E. ulmoides leaves* (TFEL) have been shown to effectively inhibit abnormal weight gain, degenerative changes in bone microstructure, and bone loss induced by estrogen deficiency in ovariectomized (OVX) rats. Of particular interest, while exerting bone-protective effects, TFEL did not induce a proliferative response in uterine tissues or other organs. Furthermore, gut microbiota analysis revealed that oral administration of TFEL significantly increased the diversity of the gut microbiota and restored intestinal microbial homeostasis in OVX rats, providing new theoretical support for microbiota-bone metabolism axis-targeted interventions in postmenopausal osteoporosis (Yin et al., 2025). Treatment with total glycosides from *E. ulmoides seeds* (TGEUS) has been demonstrated to effectively suppress OVX-induced bone loss by modulating the Notch signaling pathway. This intervention markedly enhanced the osteogenic potential of adipose-derived mesenchymal stem cells (ADSCs) in OVX rat models and improved bone formation by promoting bone matrix mineralization (Zhou and Xie, 2021). In addition, the aqueous extract of *E. ulmoides* effectively maintained the biomechanical strength and quality parameters of bone tissue by significantly inhibiting the expression of the bone turnover marker TRACP-5b (Li et al., 2016).

*In vitro* experiments and animal model studies have demonstrated that the extract significantly enhances bone metabolism homeostasis and effectively prevents bone loss. Considering the marked heterogeneity among previous findings, we conducted a meta-analysis to systematically synthesize the available evidence, thereby providing an evidence-based rationale for future clinical randomized controlled trials investigating the use of *E. ulmoides* in the treatment of osteoporosis.

## Methods

This meta-analysis followed the Preferred Reporting Items for Systematic Reviews and Meta-Analyses (PRISMA) guidelines (Liberati et al., 2009) and was prospectively registered in PROSPERO (CRD420251003546).

### Literature search strategy

We searched Chinese and English databases, including PubMed, Web of Science, Embase, Scopus, Foreign Medical Literature Retrieval Service, China National Knowledge Infrastructure (CNKI), and Wanfang Data Knowledge Service Platform. Two authors independently conducted the literature search. Database searches were conducted using a combination of keywords and Medical Subject Headings (MeSH) terms. A search was conducted from database inception to March 6, 2025, using the following combination of terms: (“Osteoporosis” OR “Osteoporoses” OR “Age-Related Osteoporosis” OR “Age-Related Osteoporoses” OR “Bone Loss, Age-Related” OR “Age-Related Bone Loss” OR “Senile Osteoporosis” OR “Post-Traumatic Osteoporosis”) AND (“Eucommiaceae” OR “Eucommia ulmoides” OR “Du-zhong” OR “Du zhong”) AND (“rats” OR “Rat” OR “Rattus” OR “*Rattus norvegicus*” OR “Laboratory Rats” OR “Norway Rats”).

### Inclusion and exclusion criteria

This study employed a randomized controlled trial (RCT) design to systematically compare the intervention effects of *E. ulmoides extract* with saline or placebo (vehicle treatment) in osteoporosis rat models. Inclusion criteria were defined as: a) rat models with successfully induced osteoporosis; b) *in vivo* experimental studies; c) clear outcome indicators with extractable data; d) randomized controlled trials (RCTs). Exclusion criteria were as follows: a) studies involving animal models with coexisting bone metabolic disorders; b) *in vitro* studies involving combination therapies or compound formulations; c) studies with duplicate data or publications; d) non-primary research types, such as conference abstracts, literature reviews, expert commentaries, or letters to the editor.

### Data extraction and quality assessment

After duplicate removal, the titles and abstracts of the remaining studies were independently screened in a double-blind manner by two researchers, excluding those that met the predefined criteria. Full-text review was conducted for studies passing initial screening to confirm their adherence to the inclusion criteria. In case of disagreements regarding study inclusion, consensus was reached through discussion or by involving a third researcher for arbitration.

Data collection was independently carried out by two researchers in a double-blind manner. The data items collected included: first author information, year of publication, methods for establishing the osteoporosis induction model, experimental subjects’ body weight and age (in months), sample size, intervention protocols, route of administration, study duration (with specified time units), and the mean and standard deviation (SD) of primary efficacy outcomes. For numerical data presented in graphical form, the GetData Graph Digitizer system (Version 2.26) was employed to digitize and reconstruct the data.

We independently applied the SYRCLE risk-of-bias tool (Hooijmans et al., 2014) to assess ten items across six domains—selection bias, performance bias, detection bias, attrition bias, reporting bias, and other biases. Studies that satisfied the criteria for each item were rated as low risk of bias, those failing to meet the criteria were rated as high risk, and studies with insufficient information were classified as unclear risk of bias. Throughout the assessment, any disagreements were resolved through discussion to ensure accuracy and consistency of the results.

### Outcome indicators

The primary outcome measure was bone mineral density (BMD). Secondary outcome measures comprised bone histomorphometric parameters—trabecular number (Tb.N), trabecular thickness (Tb.Th), trabecular separation (Tb.Sp), bone volume fraction (BV/TV), and structural model index (SMI)—and biochemical markers of bone turnover: procollagen type I N-terminal propeptide (PINP), estradiol (E2), serum alkaline phosphatase (ALP), serum osteocalcin (OC), tartrate-resistant acid phosphatase (TRACP), serum calcium, and serum phosphate.

### Statistical analysis

Data synthesis and statistical analyses were conducted using Stata SE version 18 and RevMan version 5.4 for processing and graphical presentation. Continuous variables were exported to Microsoft Excel for the calculation of means and standard deviations (SD). Heterogeneity among studies was evaluated using the I^2^ statistic; an I^2^ < 50% prompted the use of a fixed-effects model, whereas an I^2^ ≥ 50% led to the application of a random-effects model—or, where appropriate, a fixed-effects model—based on the underlying heterogeneity sources. To explore potential sources of heterogeneity, subgroup and leave-one-out sensitivity analyses were performed to assess the robustness of the findings. Publication bias was assessed by Egger’s regression test and funnel plot asymmetry, with p > 0.05 indicating the absence of significant bias. For continuous outcomes, standardized mean differences (SMD) with 95% confidence intervals (CI) were calculated, and statistical significance was set at p < 0.05.

## Results

### Retrieve results

**FIGURE 1:**
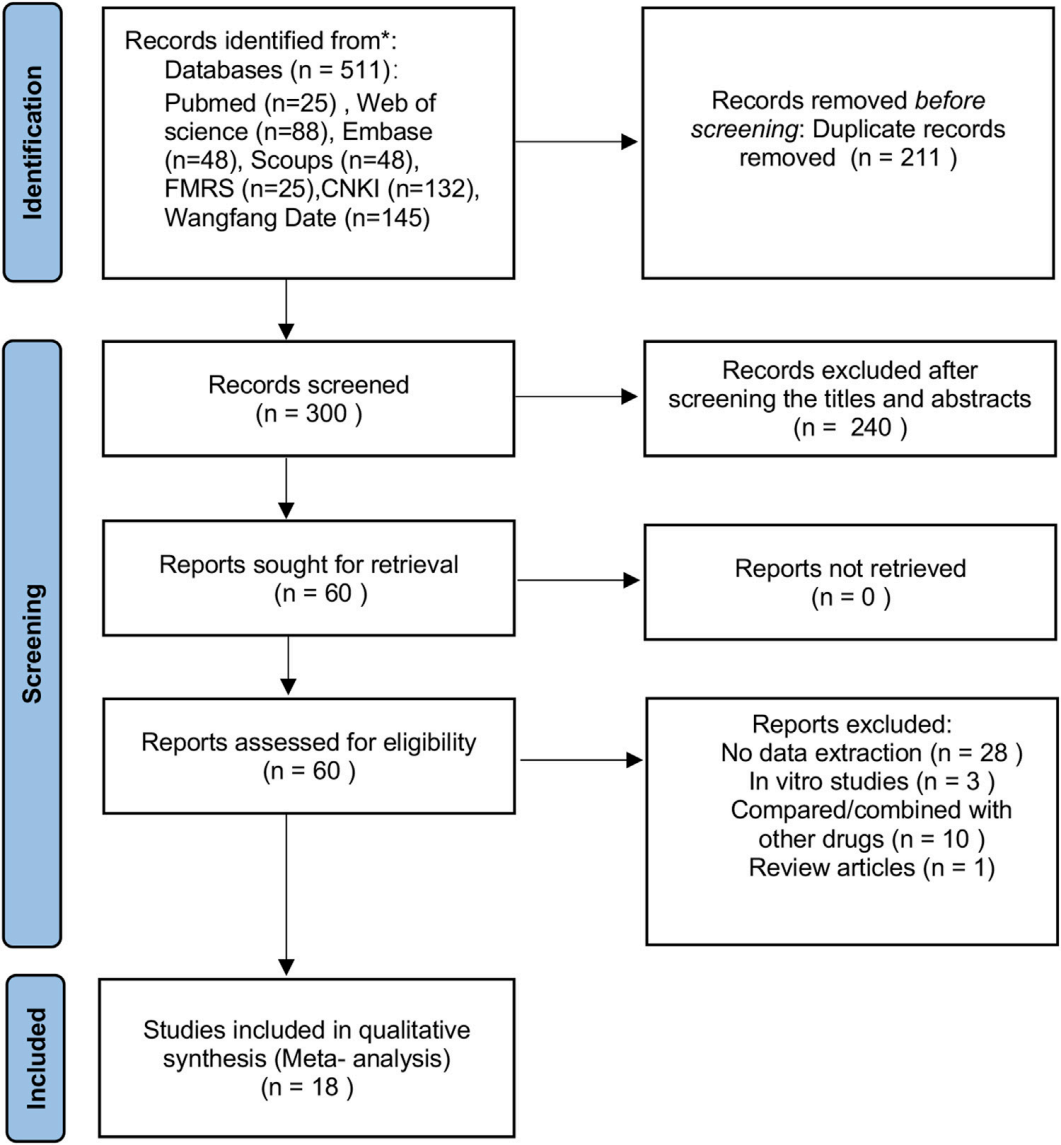
PRISMA flow chart of study selection.

The study selection process is illustrated in Figure 1. After screening seven databases, 511 records were retrieved, of which 211 duplicates were removed. Following title and abstract screening, 240 records were excluded. Sixty full-text articles were then assessed for eligibility, and 42 were excluded for the following reasons: a) 28 did not provide data on primary outcomes; b) 10 involved comparisons or co-administration with other agents; c) 3 were *in vitro* studies; and d) 1 was a review. Ultimately, 18 studies were included in the meta-analysis: 4 published in English (Yin et al., 2025; Zhang R. et al., 2009; Zhang et al., 2014; Zhou et al., 2016) and 14 published in Chinese (Du et al., 2023; Gao et al., 2016; Li S. et al., 2018; Lin, 2018; LIu et al., 2024; Liu et al., 2018; Luo et al., 2024; Luo et al., 2016; Min et al., 2022; Tong et al., 2013; Xie et al., 2022; Yang and Guan, 2023; Yuan et al., 2018; Zhang X. et al., 2009).

### Characteristics of the study

Table 1 summarizes the main characteristics of the included studies. This meta-analysis comprised 18 studies published between 2009 and 2025 examining the effects of *E. ulmoides extracts* on ovariectomy (OVX)-induced osteoporosis in rat models. Regarding extraction and purification, three studies provided detailed characterization of the active constituents, five employed ethanol extraction to isolate these constituents, one utilized distilled water extraction, and the remaining nine studies administered *E. ulmoides extracts* without specifying active constituents or extraction methods. Rats in both intervention and control groups received treatments via oral gavage, with doses ranging from 50 mg/kg/day to 6 g/kg/day administered six times per week, and study durations from 6 weeks to 200 days.

**TABLE 1:** Characteristics of the included studies.

First author	Induction of osteoporosis	Effective substance	Sample size		Intervention		Methods of administration	Duration of study
			IG	CG	IG	CG		
Liu et al. (2024)	OVX	Eucommia extract (5一HMF)	6	6	100 mg/(kg·d)	Equal physiological saline	Intragastric	12 weeks
Yuan et al. (2018)	OVX	Eucommia extract	9	7	50 mg/(kg·d)	Equal physiological saline	Intragastric	12 weeks
Luo et al. (2024)	OVX	Ethanol extract	6	6	1,080 mg/(kg·d)	Equal physiological saline	Intragastric	200 days
Luo et al. (2016)	OVX	Eucommia extract	12	12	576 mg/(kg·d)	Equal physiological saline	Intragastric	16 weeks
Liu et al. (2018)	OVX	Eucommia extract	18	18	600 mg/(kg·d)	Equal distilled water	Intragastric	8 weeks
Du et al. (2023)	OVX	Eucommia extract	7	7	2,600 mg/(kg·d)	CMC-Na	Intragastric	12 weeks
Li S. et al. (2018)	OVX	Ethanol extract	6	6	200 mg/(kg·d)	Equal distilled water	Intragastric	12 weeks
Tong et al. (2013)	OVX	Eucommia extract	15	15	6 g/(kg·d), 6 times/week	Equal physiological saline	Intragastric	12 weeks
Min et al. (2022)	OVX	Eucommia extract (Quercetin)	10	10	50 mg/(kg·d)	CMC-Na	Intragastric	8 weeks
Yang and Guan (2023)	OVX	Eucommia extract	20	20	2.76 g/(kg·d)	Equal distilled water	Intragastric	12 weeks
Lin (2018)	OVX	Eucommia extract	15	15	4 g/(kg·d)	CMC-Na	Intragastric	12 weeks
Xie et al. (2022)	OVX	Eucommia extract (Pinoresinol diglucoside)	10	10	50 mg/(kg·d)	Equal distilled water	Intragastric	6 weeks
Zhang X. et al. (2009)	OVX	Eucommia extract	20	20	330 mg/(kg·d)	Equal distilled water	Intragastric	22 weeks
Gao et al. (2016)	OVX	Distilled water extract	12	12	1,000 mg/(kg·d)	Equal distilled water	Intragastric	12 weeks
Zhou et al. (2016)	OVX	Eucommia extract(Chlorogenic Acid)	10	10	45 mg/(kg·d)	Equal physiological saline	Intragastric	12 weeks
Zhang R. et al. (2009)	OVX	Ethanol extract	10	20	500 mg/(kg·d)	vehicle	Intragastric	16 weeks
Yin et al. (2025)	OVX	Ethanol extract	8	8	200 mg/(kg·d)	Equal distilled water	Intragastric	13 weeks
Zhang et al. (2014)	OVX	Ethanol extract	10	10	80 mg/(kg·d)	Equal physiological saline	Intragastric	16 weeks

OVX, Ovariectomy; 5一HMF, 5-Hydroxymethylfurfural; CMC-Na, Carboxymethylcellulose sodium; IG, Intervention Group; CG, Control Group.

### Quality assessment result

**FIGURE 2:**
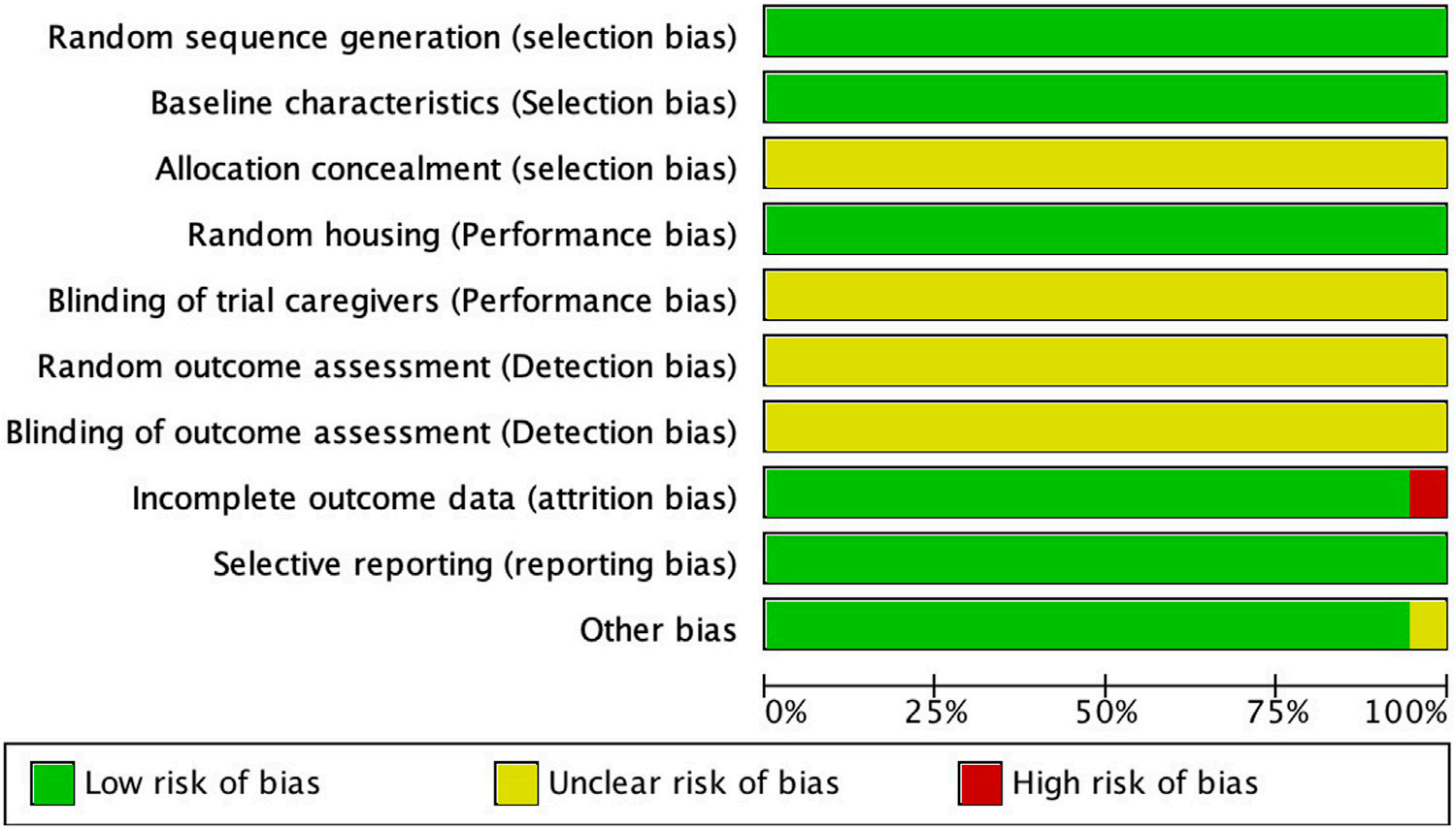
Quality of the included studies.

The risk of bias in animal studies was independently assessed using the SYRCLE risk-of-bias tool. The SYRCLE tool evaluates ten items across six domains: selection bias (random sequence generation, baseline characteristics, allocation concealment); performance bias (random housing of animals, blinding of personnel caring for the animals); detection bias (random outcome assessment, blinding of outcome assessment); attrition bias (incomplete outcome data); reporting bias (selective outcome reporting); and other bias (other sources of bias). As illustrated in Figure 2, one study was rated as high risk for attrition bias due to incomplete outcome data, and one study had an unclear risk of other biases. None of the studies reported allocation concealment, blinding of personnel, random outcome assessment, or blinding of outcome assessment. All studies adequately reported random sequence generation, baseline characteristics, and selective reporting, which were therefore rated as low risk.

### Meta-analysis

#### Bone mineral density

**FIGURE 3:**
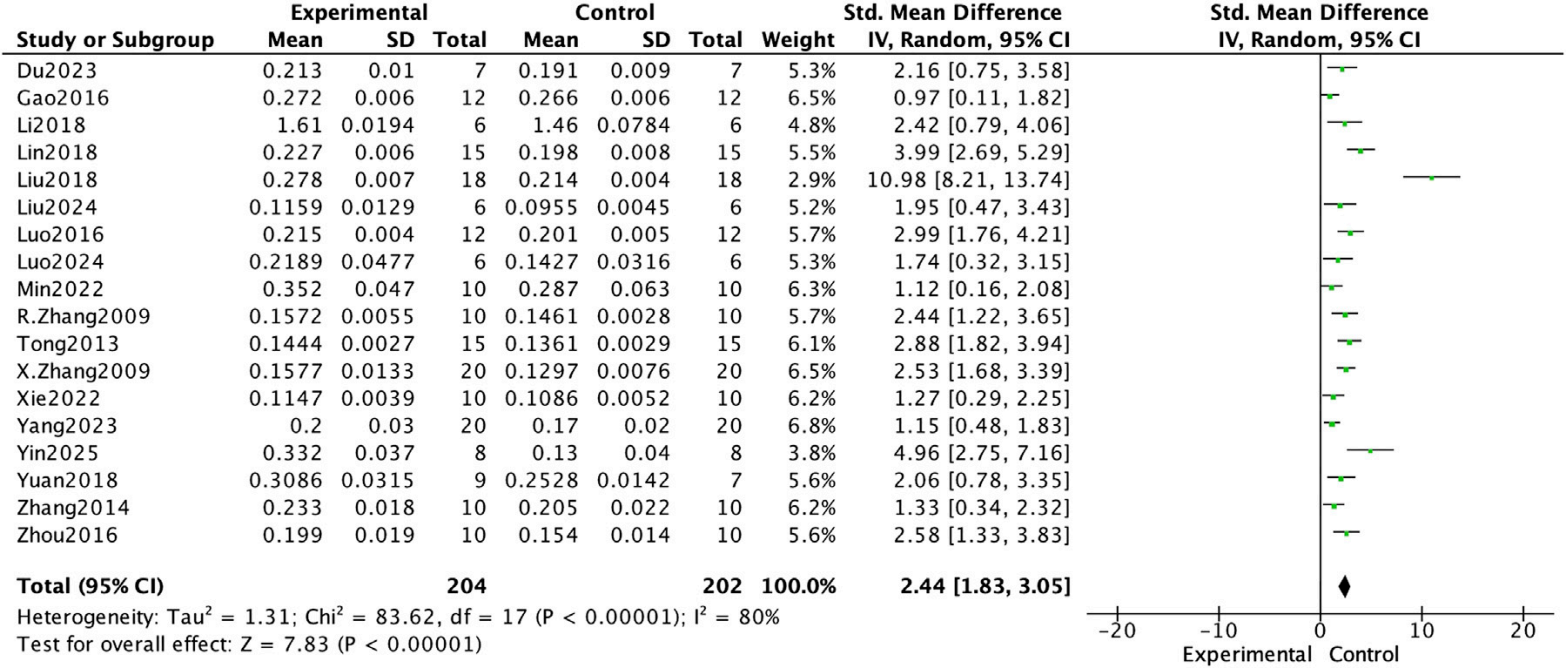
Forest plot comparing BMD between the Eucommia ulmoides group and the control group.

In this meta-analysis of bone mineral density (BMD) improvement in osteoporotic rat models, *E. ulmoides extract* was shown to exert a significant therapeutic effect. The analysis included data from 18 experiments, and as illustrated in Figure 3, BMD in the Eucommia-treated group was significantly higher than in the control group (standardized mean difference [SMD] = 2.44, 95% confidence interval [CI] 1.83–3.05; p < 0.000001). Subgroup analyses further revealed that both the dosage and treatment duration of Eucommia had significant effects on BMD enhancement, as shown in Table 2. When the Eucommia dose exceeded 400 mg/kg/day, the increase in BMD was most pronounced; similarly, treatment durations longer than 12 weeks yielded the greatest improvement in BMD. These findings suggest that *E. ulmoides extract* can effectively improve bone mineral density in osteoporotic rats under specific dosing and treatment conditions.

**TABLE 2:** Subgroup analysis of bone mineral density according to the dose and duration of Eucommia ulmoides treatment.

Subgroup	Standardized mean difference (95% confidence interval)	I^2^	p value
Dose			
≤400 mg/kg/d	2.25 [1.55, 2.94]	68	0.000
>400 mg/kg/d	2.90 [1.79, 4.01]	88	0.000
Duration			
≤12Weeks	2.08 [1.72, 2.44]	84	0.000
>12Weeks	2.33 [1.86, 2.81]	56	0.000

#### Bone histomorphometric

**FIGURE 4:**
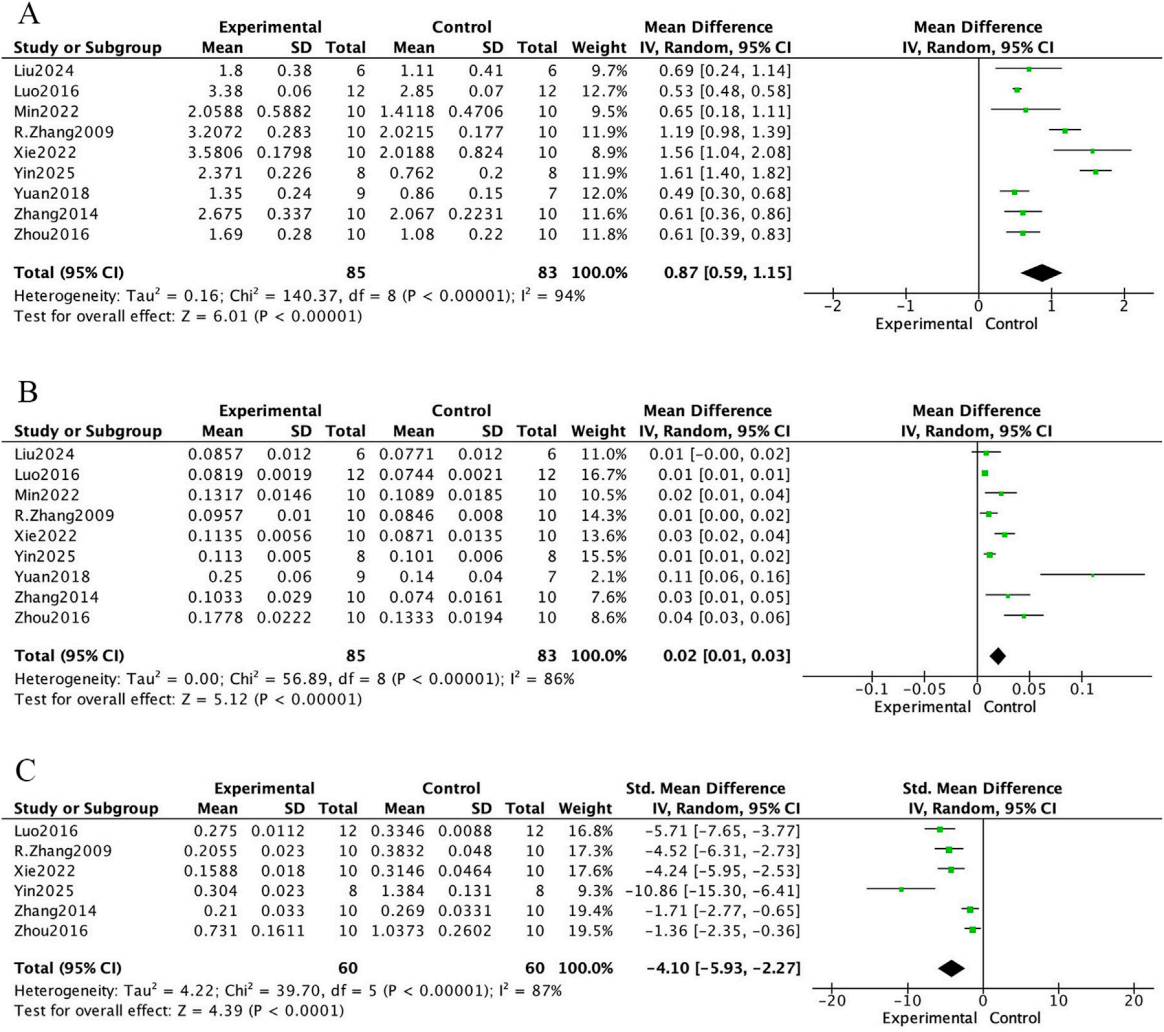
Forest plot. **(A)** Tb.N. **(B)** Tb.Th. **(C)** Tb.Sp.

**FIGURE 5:**
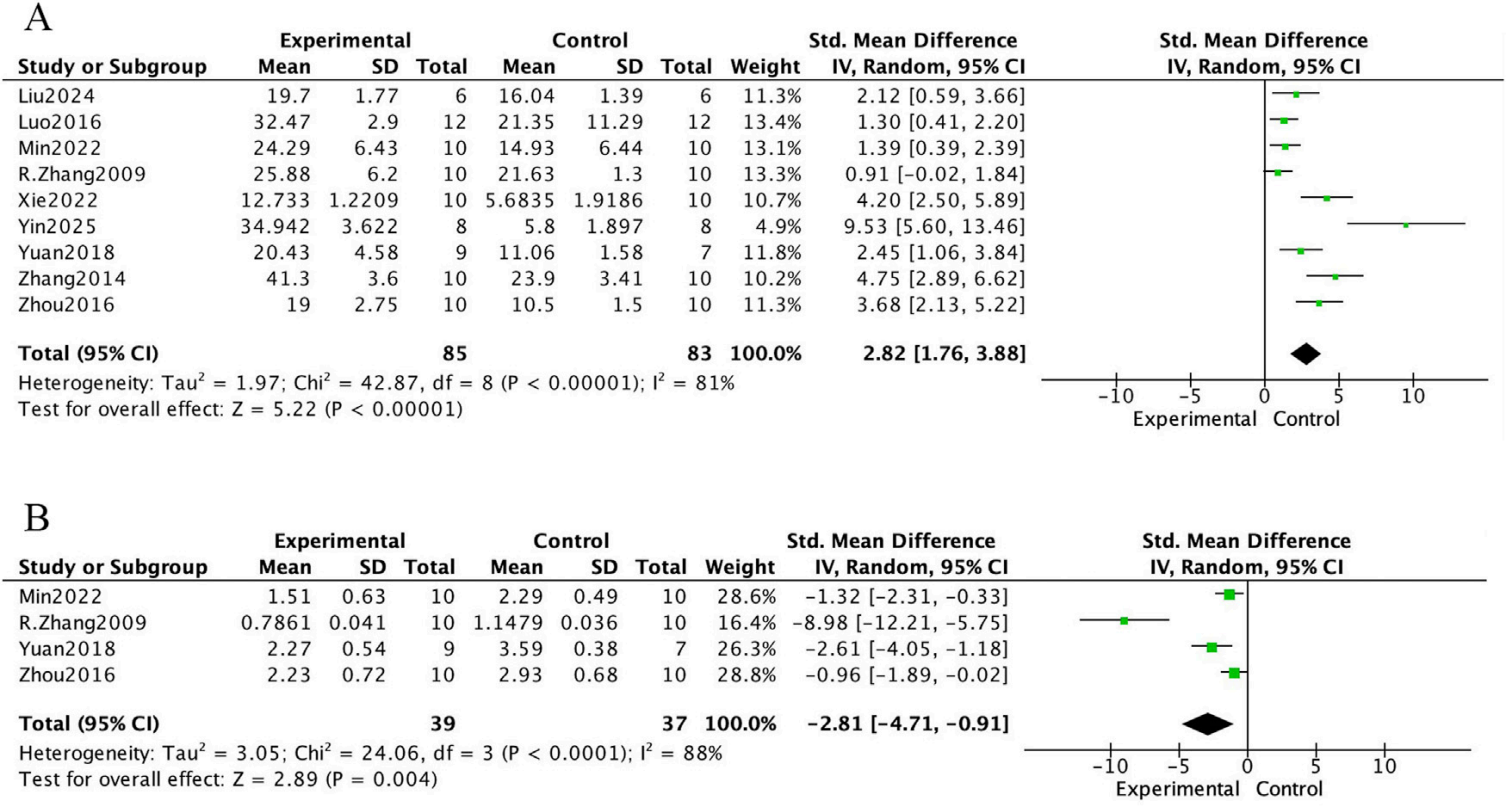
Forest plot. **(A)** BV/TV. **(B)** SMI.

The bone histomorphometric meta-analysis of *E. ulmoides extract* in osteoporotic rat models is presented in Figures 4 and 5. In Figure 4, nine studies reported that treatment with *E. ulmoides extract* significantly increased trabecular number (mean difference [MD] = 0.87; 95% CI, 0.59–1.15; p < 0.000001). Nine studies reported changes in trabecular thickness (MD = 0.02; 95% CI, 0.01–0.03; p < 0.000001). Additionally, six studies indicated that *E. ulmoides extract* reduced trabecular separation (standardized mean difference [SMD] = −4.10; 95% CI, −5.93 to −2.27; p < 0.000001). Figure 5 illustrates the effects of *E. ulmoides extract* on bone volume fraction (BV/TV) and structural model index (SMI) in osteoporotic models. Nine studies demonstrated improvement in BV/TV (SMD = 2.82; 95% CI, 1.76–3.88; p < 0.000001), and four studies reported a reduction in SMI (SMD = −2.81; 95% CI, −4.71 to −0.91; p < 0.000001).

#### Bone biochemical markers

**FIGURE 6:**
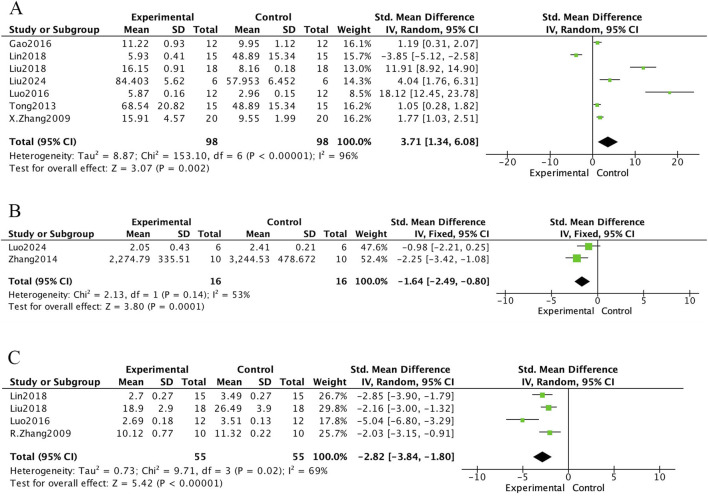
Forest plot. **(A)** E2. **(B)** TRACP. **(C)** OC.

**FIGURE 7:**
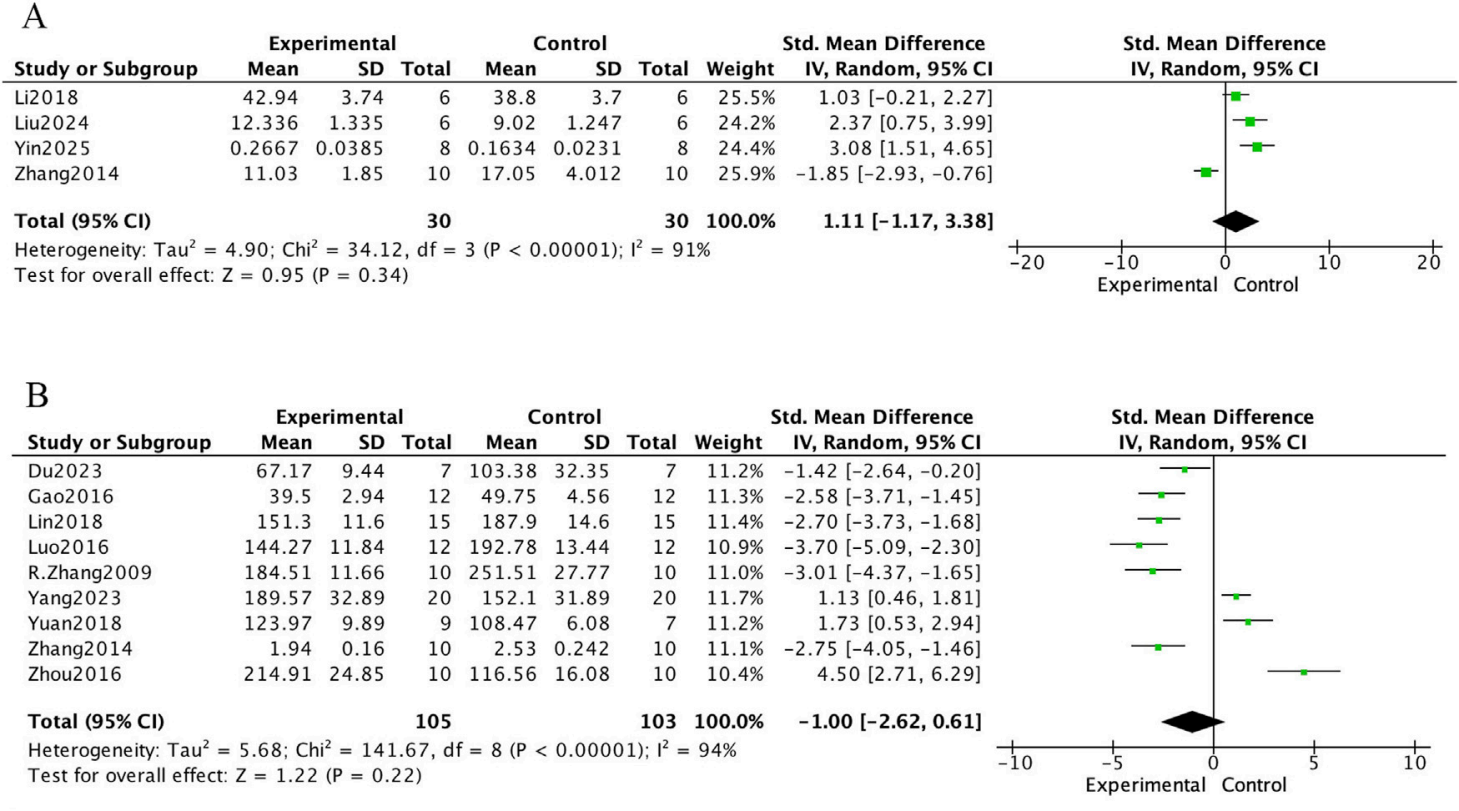
Forest plot. **(A)** PINP. **(B)** ALP.

**FIGURE 8:**
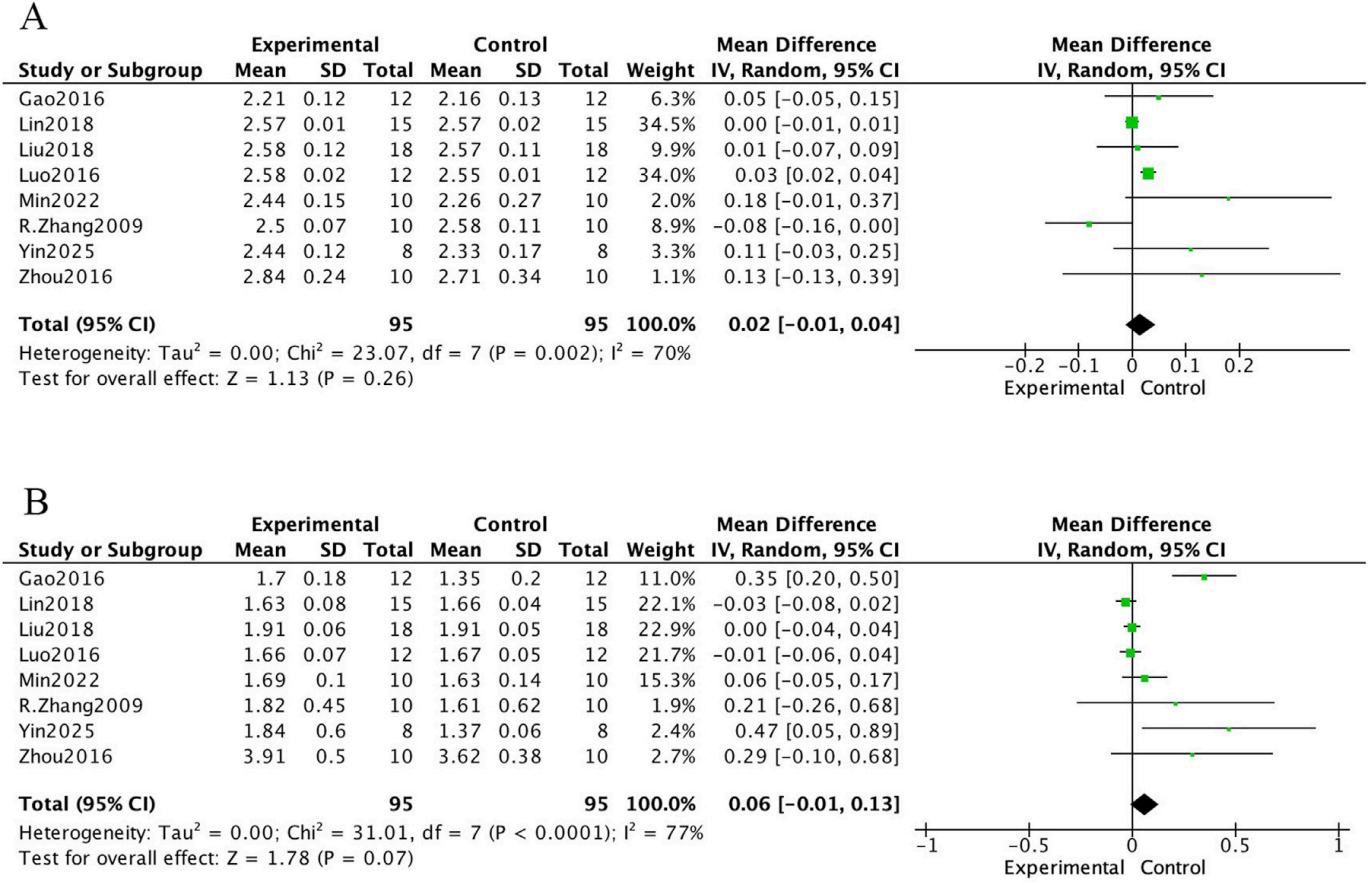
Forest plot. **(A)** Serum calcium. **(B)** Serum phosphate.

The meta-analysis of bone biochemical markers in OVX-induced osteoporotic rat models treated with *E. ulmoides extract* is presented in Figures 6–8. In Figure 6, seven studies demonstrated that Eucommia intervention significantly increased serum estradiol (E2) levels (standardized mean difference [SMD] = 3.71; 95% confidence interval [CI], 1.34–6.08; p = 0.002). Two studies reported a reduction in tartrate-resistant acid phosphatase (TRACP) levels (SMD = −1.64; 95% CI, −2.49 to −0.80; p = 0.0001), and four studies demonstrated decreased serum osteocalcin (OC) levels (SMD = −2.82; 95% CI, −3.84 to −1.80; p < 0.000001). Figure 7 depicts outcomes for procollagen type I N-terminal propeptide (PINP) and alkaline phosphatase (ALP). Four studies reported PINP after Eucommia intervention (SMD = 1.11; 95% CI, −1.17 to 3.38; p = 0.34), showing no significant effect. Moreover, nine studies showed no significant change in ALP (SMD = −1.00; 95% CI, −2.62 to 0.61; p = 0.22). In Figure 8, eight studies reported serum calcium levels (mean difference [MD] = 0.02; 95% CI, −0.01 to 0.26; p = 0.26) and eight studies reported serum phosphate levels (MD = 0.06; 95% CI, −0.01 to 0.13; p = 0.07), neither of which reached statistical significance.

### Sensitivity analysis and publication bias

**FIGURE 9:**
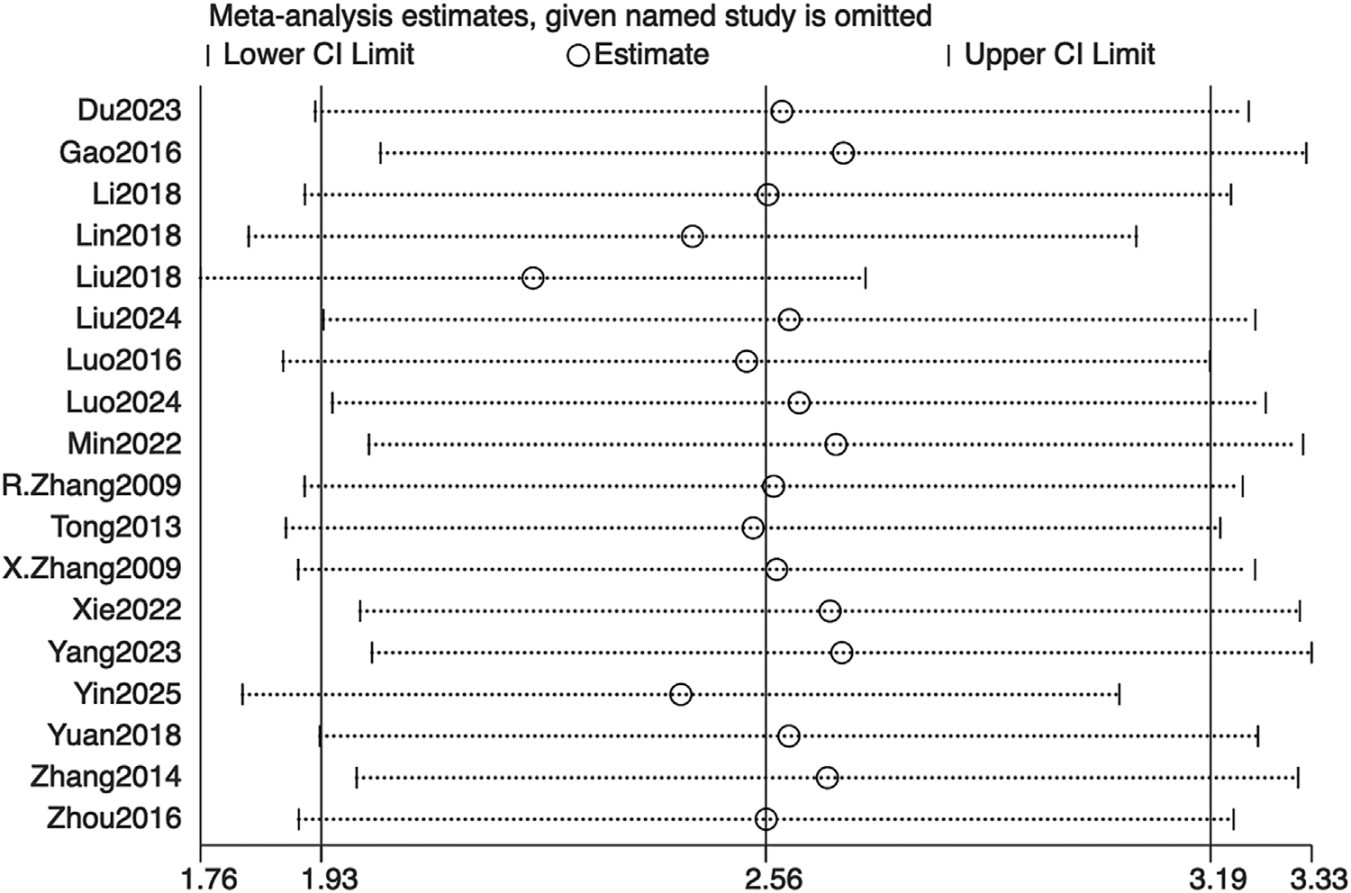
Sensitivity analysis of bone mineral density. CI: confidence interval.

Sensitivity was assessed using a leave-one-out sensitivity analysis, as illustrated in Figure 9. After sequentially omitting individual studies, the I^2^ statistic and its 95% confidence interval remained largely unchanged, indicating minimal heterogeneity and demonstrating the robustness of the meta-analysis findings. Given the relatively small and comparable sample sizes across studies, outcomes were analyzed as continuous variables. In accordance with Cochrane Collaboration guidelines, publication bias was not assessed by funnel plot or Egger’s test.

## Discussion

This meta-analysis demonstrates that *E. ulmoides extract* significantly improves bone mineral density (BMD) in osteoporotic rat models, effectively delaying bone loss. Moreover, the effect exhibits a positive correlation with both the treatment dosage and duration. Subgroup analysis revealed that *E. ulmoides* extract effectively increases BMD in osteoporotic rats at specific dosages (>400 mg/kg/d) and treatment durations (>12 weeks).

Following Total Flavonoid Extract from *E. ulmoides* (TFEL) intervention, the bone tissue microstructure of OVX rats demonstrated a significant improvement trend, with the deterioration of trabecular bone microstructural geometry and connectivity being largely prevented (Yin et al., 2025; Zhang R. et al., 2009; Zhang et al., 2014). The underlying mechanism likely involves modulation of the Osteoprotegerin/Receptor Activator of Nuclear Factor Kappa-B Ligand (OPG/RANKL) signaling pathway. The improvement in bone microstructure by *E. ulmoides* extract primarily stems from its precise regulation of the core pathway for osteoclast differentiation. Studies indicate that flavonoids within *E. ulmoides* can stably bind to specific key sites on the RANKL protein via hydrogen bonding. This action directly disrupts the RANKL-RANK interaction, mimicking the biological function of the natural inhibitor OPG (Zhang et al., 2025). By modulating the ratio of key regulatory factors within the OPG/RANKL pathway, it effectively inhibits the ligand-receptor binding of RANKL to RANK, thereby decelerating the rate of osteoclast differentiation and reducing bone resorption activity (Yin et al., 2025). This pharmacodynamic profile confirms that *E. ulmoides* extract possesses both preventive and therapeutic effects against estrogen deficiency-induced bone loss.

The observed elevation of serum estradiol (E_2_) levels by *E. ulmoides* extract reveals its non-hormone replacement regulatory mechanism. Unlike conventional estrogen therapy, the active constituent pinoresinol diglucoside in Eucommia ulmoides selectively activates estrogen receptor beta (ERβ). This enables precise modulation of bone metabolism while avoiding the risk of excessive mammary tissue proliferation (Wang et al., 2011). The reduction in Tartrate-Resistant Acid Phosphatase (TRACP) levels alongside decreased serum Osteocalcin (OC) levels reflects the extract’s role in rebalancing bone turnover. *Eucommia ulmoides* extract achieves OC normalization by promoting hydroxyapatite deposition while inhibiting abnormal degradation of the bone matrix (Li Y. et al., 2018; Schini et al., 2023). This dual regulatory effect plays a key role in improving bone microstructure and enhancing bone strength. The lack of significant changes in serum levels of Procollagen Type I N-terminal Propeptide (PINP) and Alkaline Phosphatase (ALP) suggests that the pro-osteogenic effect of *E. ulmoides* extract primarily targets the terminal mineralization stage, promoting hydroxyapatite crystal deposition, rather than the early activation of osteoblastic activity. The absence of significant fluctuations in serum calcium and phosphorus levels is attributed to compensatory regulation by the kidneys maintaining homeostasis.

*Eucommia ulmoides* contains diverse compounds including flavonoids, lignans, iridoids, phenolic acids, polysaccharides, and terpenoids. The bioactive components obtained vary significantly depending on processing methods and extraction techniques: Ethanol extraction primarily yields lignans, flavonoids, and iridoids as major active constituents, whereas water extraction predominantly yields phenolic acids and polysaccharides. The bioactive phytochemicals in *E. ulmoides Oliv extract*—including quercetin (QUE), geniposide (GEN), chlorogenic acid, Eucommia olmoides cortex polysaccharide-3 (EuOCP3), and pinoresinol diglucoside—have been demonstrated to synergistically inhibit osteoclast activity and promote osteoblast differentiation, thereby markedly delaying the progression of osteoporosis. In ethanolic extracts: Quercetin (QUE) specifically activates the nuclear factor erythroid 2-related factor 2/heme oxygenase-1 (Nrf2/HO-1) pathway, effectively mitigating iron overload–induced oxidative stress and conveying significant osteoprotective effects (Xiao et al., 2023). Geniposide (GEN) markedly suppresses dexamethasone (DEX)-induced MC3T3-E1 osteoblast apoptosis in both *in vivo* and *in vitro* models by activating the autophagy signaling pathway (Huang et al., 2022). Mechanistically, GEN’s autophagy-inducing effect is mediated via the glucagon-like peptide-1 receptor (GLP-1R)/PI3K/Akt/mTOR pathway. Notably, specific inhibition of GLP-1R expression completely abrogates GEN’s protective effect in DEX-treated MC3T3-E1 cells, underscoring the receptor’s pivotal regulatory role. Chlorogenic acid preserves bone mass homeostasis by inhibiting pathological bone remodeling, exerting a suppressive effect on bone resorption in a dose-dependent manner (Yang et al., 2023), and significantly reverses key trabecular morphometric parameters—such as BV/TV and Tb.Th—in ovariectomized (OVX) rats. This effect may involve upregulation of cyclin D1 downstream of the PI3K/Akt pathway, thereby enhancing bone marrow mesenchymal stem cell (BMSC) proliferation (R. P. Zhou et al., 2016). EuOCP3, an acidic polysaccharide isolated from the cortex of *E. ulmoides, exerts* anti-osteoporotic effects by modulating gut microbial composition and serum metabolomic profiles. Mechanistic studies reveal that EuOCP3 can stimulate bone formation by improving osteoblast differentiation via the ERK/BMP-2/SMAD signaling pathway (Song et al., 2024). Furthermore, EuOCP3 activates Nrf2 signaling, effectively mitigating oxidative stress in osteoporosis model mice and normalizing bone metabolism markers (R. P. Zhou et al., 2016).

Current first-line clinical drugs for osteoporosis, such as monoclonal antibodies and bisphosphonates, exert only singular biological effects—either promoting bone formation or inhibiting bone resorption. In contrast, *E. ulmoides* extract improves osteoporotic bone through a synergistic multi-component, multi-target, multi-pathway mechanism (Wang et al., 2022). This is evidenced by increased bone mineral density (BMD) values and statistically significant improvements across multiple bone biomechanical parameters and bone metabolism markers. Its mechanisms encompass osteoclast inhibition, osteoblast promotion, and oxidative stress modulation. Functioning as a ‘bone-immune-metabolism’ multidimensional modulator, *E. ulmoides* shows promise as a novel option for comprehensive osteoporosis management, particularly suitable for early-to-mid-stage patients with contraindications to conventional anti-osteoporotic drugs or requiring long-term intervention.

Within this meta-analysis, 9 included studies specified only ‘Eucommia ulmoides extract’ as the intervention, without detailing the specific active constituents, extraction methods, or standardization criteria. This critical information gap significantly compromises evidence transparency and may introduce unquantifiable heterogeneity. As the compositional differences arising from varying extraction processes can lead to inconsistent bioactivity, the effects on secondary outcomes—including bone histomorphometric parameters and bone biochemical markers—also varied across studies. This introduces potential bias into the pooled effect sizes and limits the extrapolation of results to specific preparations. Given the insufficient reporting of process details in the original literature and the current technical inability to retrospectively analyze the actual composition of samples in published studies, we could not statistically adjust for this heterogeneity—a common limitation in meta-analyses of herbal medicines. Therefore, the current conclusions should be regarded as a preliminary exploration of the effects of ‘broadly defined Eucommia ulmoides extract,’ rather than confirmation for a standardized product. The diversity in processing and extraction methods precisely reflects the reality of traditional Chinese medicine (TCM) clinical practice: different institutions may employ distinct standardized processes. This meta-analysis integrates this ‘real-world’ variability, demonstrating that despite process inconsistencies, Eucommia ulmoides extract consistently demonstrated positive therapeutic effects. Given this inherent heterogeneity, we recommend: 1) Future studies should strictly adhere to the Technical Guideline for Quality Research of Traditional Chinese Medicine New Drugs, Trial (Technical Guideline for Quality Research of Traditional Chinese Medicine New Drugs, Trial, 2021), reporting extraction processes and component standardization methods comprehensively and clearly; 2) Standardized extract preparations should be prioritized in clinical application.

Based on current experimental evidence, the present study supports the potential of Eucommia ulmoides as a plant-based therapeutic candidate, with its dual action of promoting bone formation and suppressing bone resorption providing novel insights into therapeutic strategies for osteoporosis. With further validation in large-scale studies and translational clinical research, this natural product is anticipated to be developed as a plant-based alternative therapy targeting specific molecular pathways.

## Strengths and limitations

This study represents the first meta-analysis to evaluate the effects of *E. ulmoides extract* in osteoporotic rat models, incorporating high-quality randomized controlled trials and providing a foundation for future clinical translation. Subgroup analyses were also performed to assess the impact of extract dosage and treatment duration on bone mineral density in these models. Nonetheless, this analysis has several limitations. First, some included studies exhibited methodological shortcomings and low quality, which may compromise the validity and reliability of the meta-analysis findings, and the widespread lack of reporting on allocation concealment and blinding (particularly outcome assessor blinding) constitutes a significant source of potential performance bias and detection bias. Second, the small sample sizes in most animal experiments may increase random error and uncertainty in the results. Moreover, the limited reporting of certain outcomes restricts comprehensive synthesis and evaluation of those endpoints. Finally, although ovariectomy partially models postmenopausal osteoporosis, interspecies differences persist, and further studies are needed to validate the translational potential of these findings in human osteoporosis.

## Conclusion

This study represents the first systematic evaluation of *E. ulmoides extract* in osteoporotic rat models, demonstrating significant, dose- and time-dependent improvements in bone mineral density (BMD), trabecular microarchitecture, and bone metabolic markers. The extract’s mechanism likely involves modulation of the osteoprotegerin (OPG)/receptor activator of nuclear factor κB ligand (RANKL) pathway, resulting in inhibited osteoclast activity and enhanced osteoblast differentiation. Despite some methodological limitations and small sample sizes in the included studies, the findings indicate distinct pharmacological efficacy against osteoporosis, supporting *E. ulmoides extract* as a plant-based therapeutic candidate. Further large-scale clinical trials are warranted to confirm its safety and efficacy and to develop innovative therapeutic strategies for osteoporosis.

## Data Availability

The raw data supporting the conclusions of this article will be made available by the authors, without undue reservation.
