# Correction: Therapeutic effects of Eucommia ulmoides extract on osteoporosis rat models: a systematic review and meta-analysis

**DOI:** 10.3389/fphar.2025.1711404

**Published:** 2025-10-22

**Authors:** Zhen Chen, Chuan Leng, Tang Rui, Chaoqun Feng, Tong Li, Yang Yu, Lei Zhong, Xiaohong Fan

**Affiliations:** Department of Orthopedics, Hospital of Chengdu University of Traditional Chinese Medicine, Chengdu, China

**Keywords:** Eucommia ulmoides oliv, osteoporosis, bone mineral density, meta-analysis, rats

In the published article, the **Equal Contribution Statement** was missing. Authors **Zhen Chen**, **Chuan Leng**, and **Tang Rui** were erroneously omitted as equal contributing authors.

The original article has been updated.

